# An Unusual Case of Spontaneous Pneumothorax Presenting as Right Lower Quadrant Pain: A Case Report

**DOI:** 10.5811/cpcem.53014

**Published:** 2026-04-29

**Authors:** Timothy P. Crowe, Patrick P. Cheatle

**Affiliations:** St. Luke’s Hospital, Department of Emergency Medicine, Bethlehem, Pennsylvania

**Keywords:** pneumothorax, appendicitis, thoracic surgery, emergency medicine, case report

## Abstract

**Introduction:**

Primary spontaneous pneumothorax generally presents with symptoms of chest pain and shortness of breath. Progression to a tension pneumothorax results in a medical emergency. Rare presentations with abdominal pain are possible and must be considered to expedite appropriate treatment of pneumothorax.

**Case Report:**

We report a case of a 21-year-old male with primary spontaneous pneumothorax who initially presented to the emergency department with right lower quadrant abdominal pain. History and physical exam were suggestive of acute appendicitis. A large right pneumothorax was incidentally found on computed tomography.

**Conclusion:**

This case highlights unusual presentations of pneumothorax. Emergency physicians should consider atypical presentations of chest pathology such as pneumothorax in patients presenting with symptoms consistent with an acute abdomen.

## INTRODUCTION

Primary spontaneous pneumothorax is a potentially life-threatening condition that must be recognized expeditiously. It typically presents with symptoms of pleuritic chest pain and/or shortness of breath; however, in rare cases patients present with symptoms suggestive of an acute abdomen. We report an unusual case of primary spontaneous pneumothorax in a 21-year-old male who presented to the emergency department (ED) with symptoms mimicking acute appendicitis.

## CASE REPORT

A 21-year-old male presented to the ED for evaluation of right lower quadrant abdominal pain since shortly after waking the morning of presentation. The pain was sudden in onset, “sharp,” constant, and worse with attempted oral intake. He was walking when the pain started. He spoke with his mother, a healthcare professional, who advised him to go to the ED for probable appendicitis. He had no significant medical, surgical, or social history.

Review of systems was positive for abdominal pain and mild anorexia but was otherwise negative. Vital signs were unremarkable except for mild tachycardia at 110 beats per minute. Temperature was 98.1 °F, respiratory rate was 18, blood pressure was 143/80 millimeters of mercury, and oxygen saturation was 97% on room air. The patient was a thin but otherwise normal-appearing male. Breath sounds were auscultated bilaterally. Abdominal examination was significant for right lower quadrant tenderness but no rebound or guarding. Physical exam was otherwise unremarkable.

**Image 1:**
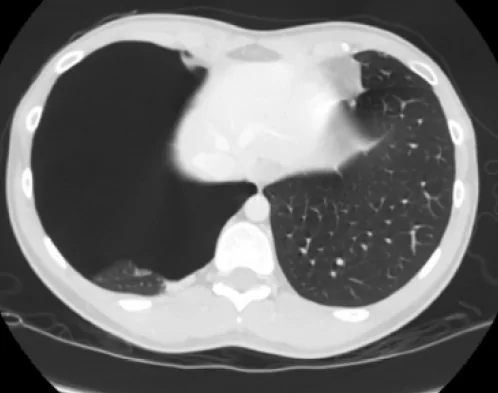
Computed tomography demonstrating incidental right-sided pneumothorax in a thin young man.

**Image 2:**
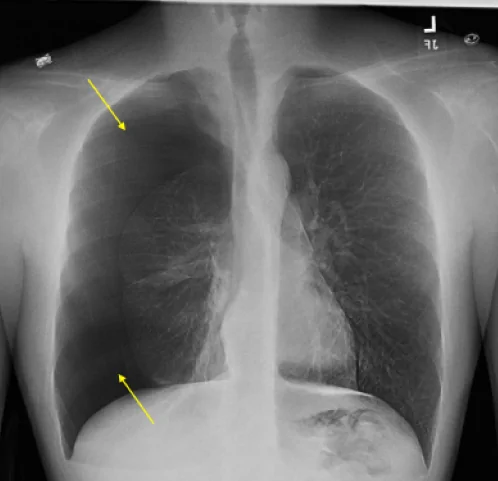
Chest radiograph demonstrating large right-sided pneumothorax.

Bloodwork included a complete blood count, complete metabolic panel, and lipase. No significant abnormalities were found. Electrocardiogram showed normal sinus rhythm with nonspecific T-wave changes. The patient declined pain medications. He continued to endorse right lower quadrant abdominal pain during his ED stay, and tachycardia resolved spontaneously. Computed tomography abdomen and pelvis showed no acute abdominopelvic pathology. Incidentally, there was a large right pneumothorax with significant atelectasis of the right lung (Image 1). A chest radiograph (CXR) was then taken (Image 2).

Thoracic surgery was consulted per patient/family request. A percutaneous 12 French chest tube was placed at bedside without incident or complication. Several days later the patient was taken to the operating room for right video-assisted thoracoscopic surgery pleurodesis and right apical blebectomy. On hospital day seven, the chest tube was removed, and he was discharged to home. Post-removal CXR showed no pneumothorax. The patient was stable at two-week follow-up visit.

## DISCUSSION

A primary spontaneous pneumothorax can occur with the rupture of subpleural air sacs called blebs. This leads to an air leak into the pleural space and eventual lung collapse. Risk factors include being a tall, thin male or having a smoking history or history of lung disease.^1^ Pneumothorax can become life-threatening if it progresses to a tension pneumothorax. Therefore, prompt diagnosis is necessary. Primary spontaneous pneumothorax was not initially high on the differential in this case. The patient did not complain of shortness of breath or chest pain. Additionally, breath sounds were auscultated bilaterally; however, these sounds could have been transmitted from the collapsed or contralateral lung.

*CPC-EM Capsule*

What do we already know about this clinical entity?

*Pneumothorax is a potentially life-threatening condition that presents typically with pleuritic chest pain or dyspnea. It classically presents in tall, thin males.*

What makes this presentation of disease reportable?

*This report describes a rare presentation of pneumothorax as right lower quadrant abdominal pain.*

What is the major learning point?

*Pneumothorax can atypically present as abdominal pain.*

How might this improve emergency medicine practice?

*Physicians should be aware of non-classical presentations of pneumothorax.*

While primary spontaneous pneumothorax generally presents with chest pain or shortness of breath, rare cases have been documented where the patient presents with symptoms of acute abdominal pain. Traditionally, hemopneumothorax^2^ and primary spontaneous pneumothorax^3^ can present as right upper quadrant pain suggesting gallbladder disease. There are also several noted cases of it presenting as epigastric pain mimicking pancreatitis.^4^,^5^,^6^

In this case, the patient demonstrated lower abdominal pain mimicking acute appendicitis. Several possible mechanisms of referred pain have been previously discussed.^5^ These include diaphragm depression due to tension pneumothorax, pleural effusion causing upper quadrant pain, or the collapsed lung pulling upward on the pulmonary ligament, which interferes with the diaphragm. Diaphragmatic irritation of the phrenic nerve, (C3–C5) can refer pain to the shoulder (Kehr sign) and sometimes produce upper abdominal pain; however, right lower quadrant pain would not be expected. In this patient, the possible etiology of pain could have been secondary to inflammation of the peripheral portions of the diaphragm and their innervation. The anterior branches of the thoracic intercostal nerves (T7–T12) can refer pain to the lower abdominal dermatomes. The thoracoabdominal nerves originate from the ventral rami of the T7 to T12 spinal nerves.^7^,^8^ Although mechanisms of abdominal pain from primary spontaneous pneumothorax are limited, diaphragmatic irritation could explain the presentation in this patient.

## CONCLUSION

Primary spontaneous pneumothorax can present with symptoms suggesting an acute abdomen, specifically acute appendicitis. Emergency clinicians should consider primary spontaneous pneumothorax on the list of differential diagnoses in a patient presenting with lower abdominal pain.
